# Automated generation of massive image knowledge collections using Microsoft Live Labs Pivot to promote neuroimaging and translational research

**DOI:** 10.1186/2043-9113-1-18

**Published:** 2011-07-15

**Authors:** Teeradache Viangteeravat, Matthew N Anyanwu, Venkateswara Ra Nagisetty, Emin Kuscu

**Affiliations:** 1 Clinical and Translational Science Institute University of Tennessee Health Science Center, Memphis, TN 38163, USA

## Abstract

**Background:**

Massive datasets comprising high-resolution images, generated in neuro-imaging studies and in clinical imaging research, are increasingly challenging our ability to analyze, share, and filter such images in clinical and basic translational research. Pivot collection exploratory analysis provides each user the ability to fully interact with the massive amounts of visual data to fully facilitate sufficient sorting, flexibility and speed to fluidly access, explore or analyze the massive image data sets of high-resolution images and their associated meta information, such as neuro-imaging databases from the Allen Brain Atlas. It is used in clustering, filtering, data sharing and classifying of the visual data into various deep zoom levels and meta information categories to detect the underlying hidden pattern within the data set that has been used.

**Method:**

We deployed prototype Pivot collections using the Linux CentOS running on the Apache web server. We also tested the prototype Pivot collections on other operating systems like Windows (the most common variants) and UNIX, etc. It is demonstrated that the approach yields very good results when compared with other approaches used by some researchers for generation, creation, and clustering of massive image collections such as the coronal and horizontal sections of the mouse brain from the Allen Brain Atlas.

**Results:**

Pivot visual analytics was used to analyze a prototype of dataset Dab2 co-expressed genes from the Allen Brain Atlas. The metadata along with high-resolution images were automatically extracted using the Allen Brain Atlas API. It is then used to identify the hidden information based on the various categories and conditions applied by using options generated from automated collection. A metadata category like chromosome, as well as data for individual cases like sex, age, and plan attributes of a particular gene, is used to filter, sort and to determine if there exist other genes with a similar characteristics to Dab2. And online access to the mouse brain pivot collection can be viewed using the link http://edtech-dev.uthsc.edu/CTSI/teeDev1/unittest/PaPa/collection.html (user name: tviangte and password: demome)

**Conclusions:**

Our proposed algorithm has automated the creation of large image Pivot collections; this will enable investigators of clinical research projects to easily and quickly analyse the image collections through a perspective that is useful for making critical decisions about the image patterns discovered.

## Background

Recent research in laboratory science and clinical trial studies has given rise to the generation of massive neuro-images and clinical image collections of very high-resolution. Thus in order to deliver massive collections so that researchers can fully interact, share, and filter image collections, an online, real-time research collaborative approach has become a very key challenge. With the recent expansion of browsing capabilities and increased network performance [1,2], delivering massive image collections has become feasible for translational researchers and clinician-scientists to analyze, interpret and even possibly diagnose from these distributed networked image collections. Given these improvements in recent modern web service technologies, a basic component to be considered when developing these distributed image portals for viewing massive image collections is the ability to efficiently interact with and effectively search large amounts of data to answer multi-dimensional analytical queries and its augmentation with pertinent experiential knowledge. The Microsoft Live Labs Pivot [3] provides a unique alternative way of viewing massive information and allows users to discover any recognizable patterns, thus offering the potential for exploring new ideas that can be used to test the research hypotheses and promote translational research. The Pivot collections use DeepZoom [4] technology and Silverlight [5] browser capability to display high-resolution image collections, which is a very fast and smooth zooming technology that provides user with a quick way to navigate through high-resolution images in multiple zoom levels.

In this article, we defined the minimum set of requirements necessary to implement such an automated process for Pivot image collections [6,7]. The large sets of images were obtained from the Allen Brain Atlas [8], a genome-wide database resource of full colour, high-resolution gene expression patterns in the mouse brain [9]. The Pivot collections were deployed in a Linux CentOS 5 2-dual core with 2.26 GHz Xeon processors (Dell R610) running Apache web server [10]; Graphics::DZI [11] is a Perl module [12] that can be used in creating and generating titles from a given collection of images but in this study Python modules [4,6,7] were used. The images are generated like the tree in the DeepZoom [4] pyramid, thus making it possible to be expanded or zoomed to yield high-resolution zoom levels. The Pivot collections were tested precisely on various operating systems like Windows 7, Vista and Mac OS X. In addition to Pivot collections, this discussion will include the full benefits of DZI generation, which are expansions and implementation of analytic and statistical functions that will extract meaningful information from Pivot collections.

A lot of effort has been expended in developing web-based image management systems which offer the potential to access image collections for research clinician-scientists [13]. Several centers and National Institutes of Health (NIH) have invested heavily in individual image-data storage and retrieval systems. Notable among them are the National Center for Research Resources' "Biomedical Informatics Research Network" (BIRN) and the National Cancer Institute's "cancer Biomedical Informatics Grid" (caBIG) [14]. The BIRN system [15] extracts/retrieves and then transmits images from a source, while caBIG manages oncology and radiology images from multiple sources through its web servers [14]. These systems encourage and foster collaboration among individuals and research groups. The Allen Mouse and Human Brain Atlas [8] provide an interactive, genome-wide image database of gene expression as a web-based resource to present a comprehensive online platform for the exploration of mouse and human brain research [8]. The BrainMaps is an NIH-funded project that provides an online interactive zoomed high-resolution digital brain atlas of massive scanned brain structure images in both primate and non-primate serial sections for research and didactic settings [16].

The Mouse Brain Library (MBL) provides massive image collections of mouse brain structure that consists of the most comprehensive sets of recombinant inbred strains, including BXD, LXS, AXB, BXH, and CXB for studies of the genetic control, function, and behaviour [9]. The Web Quantitative Trait Loci (QTL) provides a collection of images from 200 well defined strains of mice, 2200 cases, 8800 Nissl-stained slides, and about 120,000 coronal and horizontal sections of the mouse brain to support deep understanding on the genetic variability axis [17]. The Surface Management System (SuMS) database consists of large numbers of complex surface-related datasets of the cerebral cortex that many believed to be human functions which provide connectivity for learning, emotion, sensation, and movement [18]. The neuroscience community can access SuMS for searching and federating meta-information across all the datasets using a Web interface version (WebSuMS). The Gene Expression Nervous System Atlas (GENSAT) database is sponsored by National Institute of Neurological Disorders and Stroke (NINDS) [19]. The GENSAT is well-constructed and positioned to act as a database that contains large collections for a gene expression atlas of the central nervous system of the mouse based on bacterial artificial chromosomes (BACs) [20].

The National Cancer Institute of "National Biomedical Imaging Archive" (NBIA) provides an online image repository tool to access imaging resources that aims to improve the use of imaging in increasing the efficiency of imaging cancer detection, diagnosis, therapeutic response, and improved clinical decision-making support [21]. Notable projects using NBIA database are the Reference Image Database to Evaluate Response (RIDER) [22], Lung Image Database Consortium (LIDC) [23] and Virtual Colonoscopy Collection [13]. The RIDER is a collaborative pilot project sponsored by NCI that provides a resource of full-chest DICOM CT scanned images for the patient response to therapy in lung cancer treatment. The LIDC provides a Web accessible platform that consists of low-dose helical CT scan collections for lung cancer in adult patients. The International Consortium for Brain Mapping (ICBM) is a web interface for an anatomically-labelled brain database sponsored by NCI [16]. The neuro-imaging and related clinical data can be accessed by searching through a user-friendly environment called LONI Image Data Archive (IDA) [24]. The IDA is currently used for many neuroscience projects across North America and Europe for Magnetic Resonance Imaging (MRI), Positron Emission Tomography (PET), Magnetic Resonance Angiogram (MRA) and Diffusion Tensor Imaging (DTI).

Finally, several image-data management techniques with varying levels of complexity are available to related clinical researchers. The Java-based remote viewing station JaRViS was an early example of a medical image viewing and report generating tool that exploited local-area network systems for web-based image processing of diagnostic images generated through nuclear medicine [13]. Kalinski et al. introduced virtual 3D microscopy using JPEG2000 for the visualization of pathology specimens in the Digital Imaging and Communications in Medicine (DICOM) format to create a knowledge database and online learning platforms [25]. Kim et al. proposed the Functional Imaging Web (FIWeb) [26]. The FIWeb is a web-based medical image data processing and management system that uses Python and JavaScript for rendering a graphical user interface (GUI); it also uses Java Applets for development of online image processing functions. The creation of a massive data-image collection with bioinformatics functionality eliminates the problem that is encountered in querying and searching a large image set of data. It also enhances data transmission and collaboration.

## Methods

### Pivot Collection Requirements

The minimum set of requirements needed to run Pivot collections is as stated below:

• Collection.cxml -The collection extensible markup language (XML) consists of a set of rules to describe structured data to be displayed in Pivot collection. The CXML file contains the set of categories and types associated with it. The types are String, LongString, Number, Date, Time and Link that describe the majority of the information associated with the individual images in the collection. The automation of a process in generating CXML file from a given set of images is described in section 3.1.

• Collection.xml -This XML contains the unique set of identifications (ID) and the size of images (i.e., width and height) that are assigned to an individual image along with zoom levels of information. Collection.xml is automatically created when we run the "deepzoom" function in python to subdivide high-resolution images into various zoom levels. The "deepzoom" function in python can be downloaded at [27].

• Python Imaging Library (PIL) [28] - PIL provides image processing functionality and supports many file formats. We adopted the PIL version 1.1.6 (python-imaging-1.1.6) to work in conjunction with Python Deep Zoom Tools for Pivot collections.

• Python Deep Zoom Tools -The deep-zoom-tool version 0.1.0 [27] was adopted to run subdividing high-resolution image into various zoom levels described in Collection.xml.

• Collection.html -This hypertext mark-up language (html) file contains necessary information to run the Silverlight browser capability to display high-resolution image collections in client user browser.

• Silverlight.js -The JavaScript file uses Silverlight browser capability. It can be downloaded from Microsoft Live Labs Pivot website [5].

• PivotSimpleDemo.xap -This is a compiled file format that renders the graphical user-friendly interface (GUI). It is a Microsoft Silverlight [5] application that was developed in-house which is used as a Pivot viewer.

• Collection files -This contains a set of image collections in various zoom levels indicated as dzi format. The dzi format is the deep zoom file format obtained from Python Deep Zoom Tools.

### Automation of the Process of Creating Collection XML (CXML)

**Figure 1:**
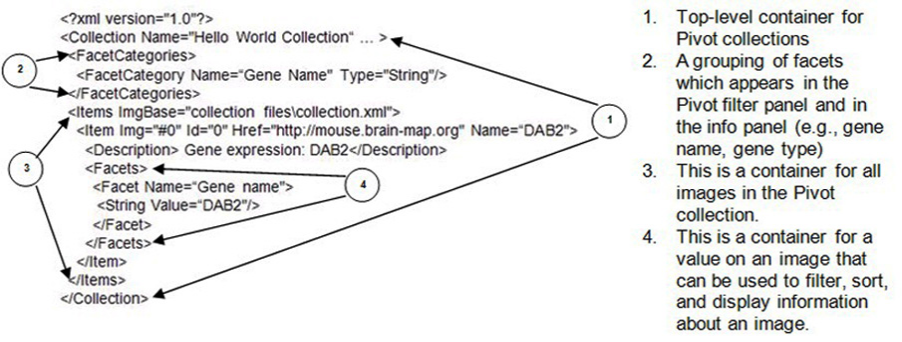
**Example of a simple collection with only one item (DAB2 gene)**. Example of a simple collection with only one item (DAB2 gene).

We have automated the process of creating collection XML (CXML) with the use of XmlWriter Class [29] which is written in Hypertext Pre-processor (PHP). The PHP is a dynamic language that is a widely-used for Web interface and application development purposes. The structure of CXML is quite simple. Below example (See Figure 1) specifies a simple collection with the only one item.

### Pivot Collection Architecture

The Pivot collection architecture is comprised of two main components that provide the ability to create a set of dzi formats from a large number of high-resolution images through the DeepZoom

**Figure 2:**
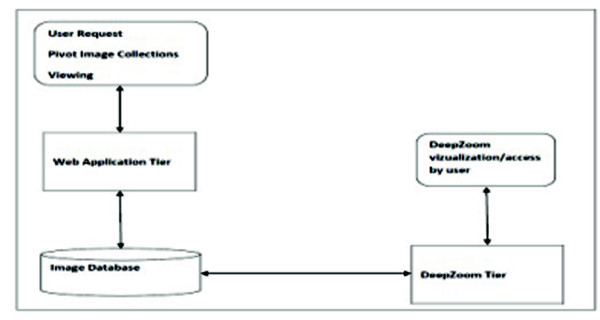
**Modify Pivot Collection Architecture**. A modified architecture showing Pivot collections.

Tier and view Pivot image collections using the Web Application Tier (See Figure 2). The image collections are stored in the image database.

### DeepZoom Tier

The DeepZoom Tier is responsible for detecting a collection request from a user through the web application tier. It is used in processing, creating, and hosting the pivot collections. The technologies necessary to run the DeepZoom Tier includes PHP, Python efficiently configured with Python Image Library, MySQL database, deep-zoom library and the Apache web-server. The deep-zoom server is comprised of two main components that provide the ability to work as an intercommunicating autonomous system and perform their respective functions.

**Figure 3:**
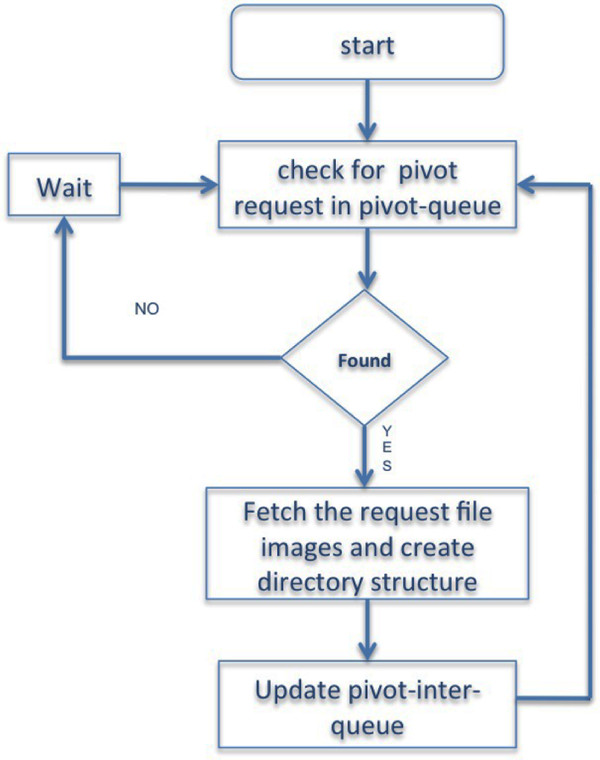
**Modify Apache directive to handle deep zoom script in python**. Flowchart showing the migration and structuring component of the algorithm.

**Figure 4:**
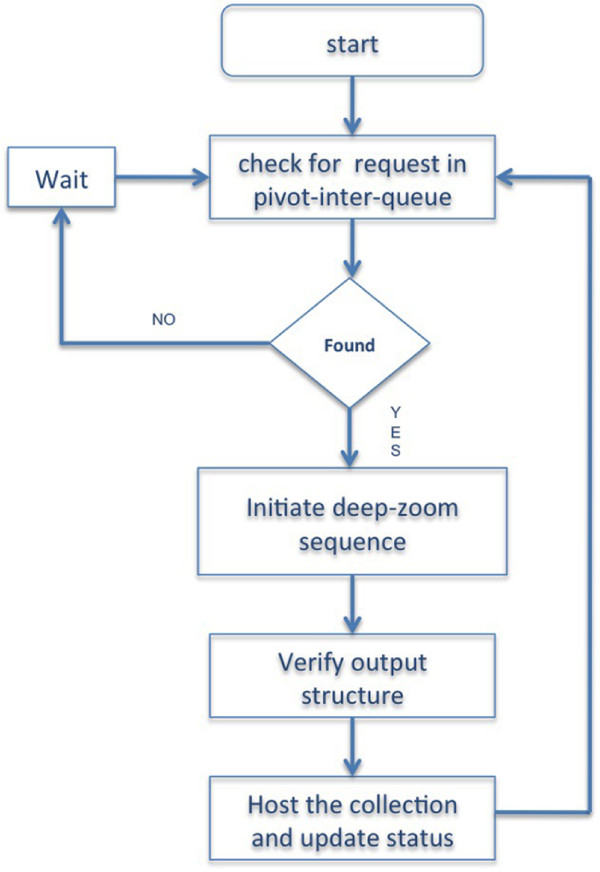
**Migration and structuring component flowchart**. Flowchart showing Pivot creation, verification and hosting.

• Migration and structuring component: This component fetches one pivot-request file each time from the pivot request queue, migrates the images from the image datasets and creates the appropriate file structure required by the pivot collection (See Figure 3).

• The Pivot creation, verification and hosting component is composed of four main steps as stated below:

1. Fetch one pivot-request from the pivot-inter-queue.

2. Create deep-zoom image partitions.

3. Create CXML, XML file and the appropriate directory structure required for pivot hosting specifications.

4. Host the collection on the local web-server (See Figure 4).

**Figure 5:**
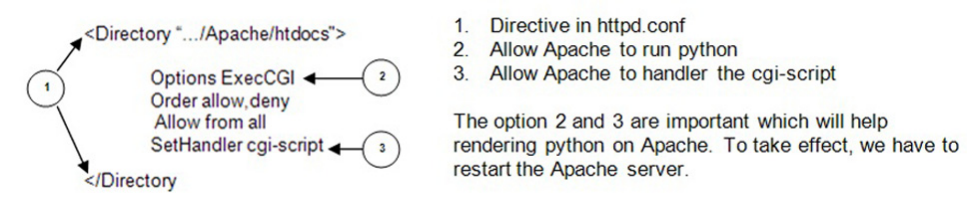
**Pivot creation, verification and hosting component**. Apache directive that handles deep zoom script in python environment.

The fundamental function of the DeepZoom Tier is to accept the client-side requests and then generate a set of dzi formats along with information about zoom levels from a given set of high-resolution images. The communication channel (C2) between the Web Application Tier and DeepZoom Tier uses Asynchronous JavaScript and XML (Ajax). In this case, we use Ajax to link PHP and Python Deep Zoom. To run python on Apache, we have to map common gateway interface (CGI) file extensions to handlers by un-commenting "AddHandler cgi-script.cgi" under the "httpd.conf" file. The "httpd.conf" is the Apache configuration file. We set the permissions of the root directory, or the directory which contains the python files (See Figure 5).

### Web Application Tier

**Figure 6:**
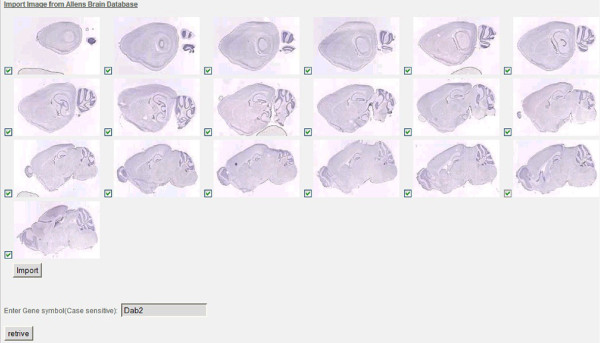
**Pivot Collection Architecture**. Pivot collection architecture using the mouse brain gene as an example.

**Figure 7:**
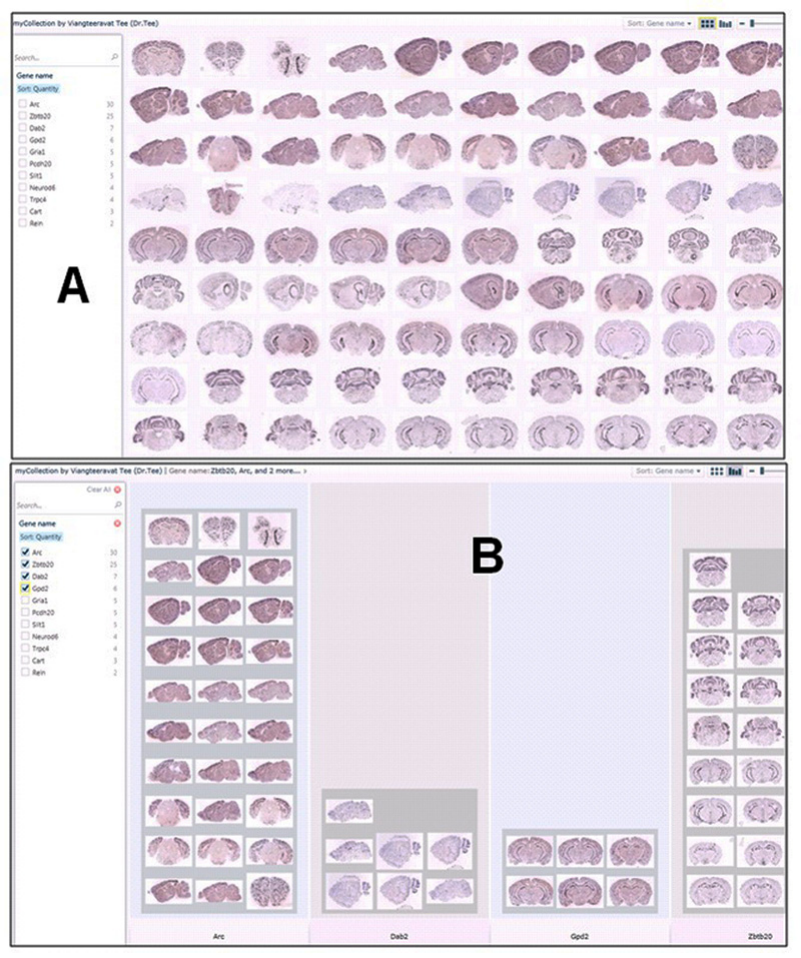
**Pivot collections. A. Forest view of gene expression maps for 11 gene symbols. B. Tree view of gene expression maps for 4 gene symbols**. Pivot collections. A. Forest view of gene expression maps for 11 gene symbols. B. Tree view of gene expression maps for 4 gene symbols.

**Figure 8:**
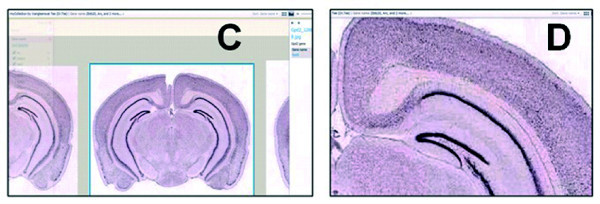
**Pivot collections C. Gpd2 -gene expression map in hippocampus (coronal view). D. Progressively deep zoom level (Gpd2)**. Pivot collections C. Gpd2 - Gene expression map in hippocampus (coronal view). D. Progressively deep zoom level (Gpd2).

The Web Application Tier consists of three main components that provide an interface to the mouse brain image database, python imaging library and deep zoom tools. The components are as follows;

• The Application Programming Interface (API). The purpose of the API is to enable the user to cluster and classify mouse brain images for a given gene symbol from the Allen Brain Atlas (see Figure 6). The communication channel (C1), which is the web browser, provides users with the ability to conduct real-time searches of related research images for Pivot collection. As depicted in Figure 6, the set of mouse brain images in the sagittal view are retrieved through gene symbol query submitted by the user. These images represent gene expression maps for the mouse brain using high-throughput procedures for in situ hybridization.

• Creation of Pivot image collections from a given set of images. The Web Application Tier summarizes the total set of images and sends them to the DeepZoom Tier. The dzi generation process takes place in queue scheduling priority. Once the process is completed, the DeepZoom Tier will be sending an auto response back to the Web Application Tier. A Linux CentOS 5 2-dual core 2.26 GHz Xeon processors (Dell R610) running Apache was deployed to run the process of dzi generation at the DeepZoom Tier. We intend to implement this on a Linux Centos 5 8-core system (Dell R610) to handle higher volume of collection requests.

• Display Pivot image collections. The Pivot image collections are shown in Figure 7 and Figure 8.

## Results

**Figure 9:**
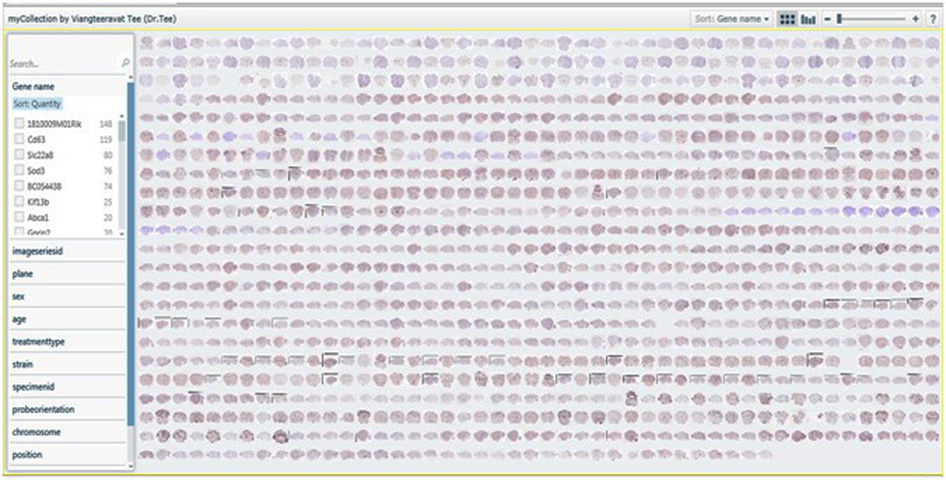
**Gene's Broad view**. Broad view of genes pivot collection.

**Figure 10:**
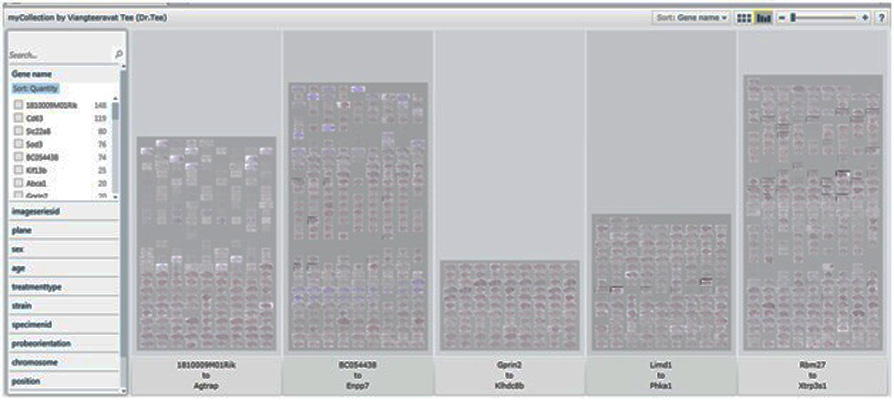
**Classification of genes**. Pivot collection showing classification of genes.

**Figure 11:**
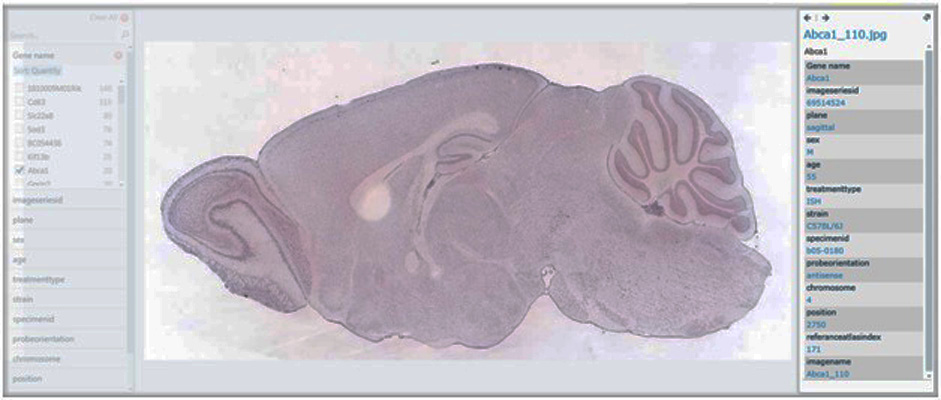
**Abca1 gene**. Broad view of Abca1 gene in the Pivot collection.

**Figure 12:**
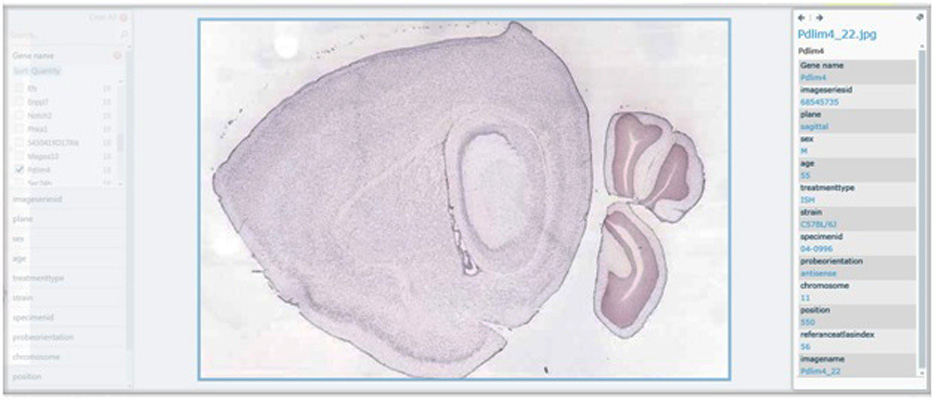
**Klhdc8b gene**. Broad view of Klhdc8b gene in the Pivot collection.

**Figure 13:**
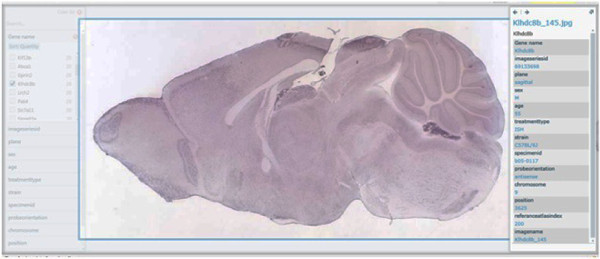
**Pdlim4 gene**. Broad view of Pdlim4 gene in the Pivot collection.

**Figure 14:**
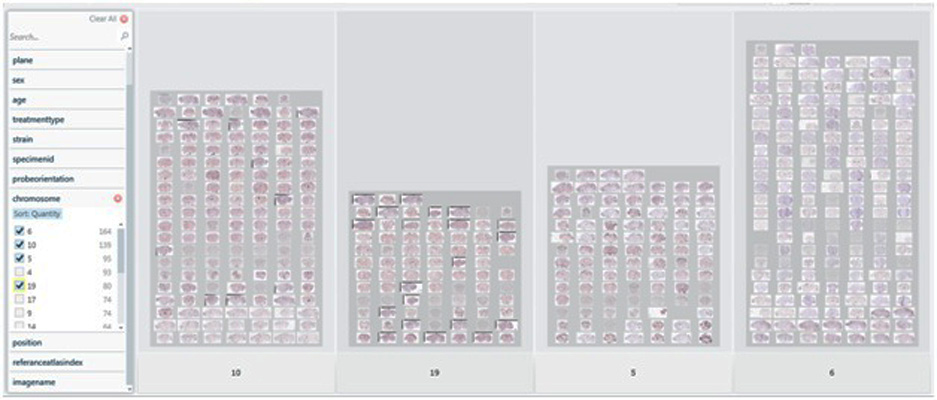
**Classification of genes based on chromosome metadata**. Pivot collection showing classification of genes based on chromosome metadata.

**Figure 15:**
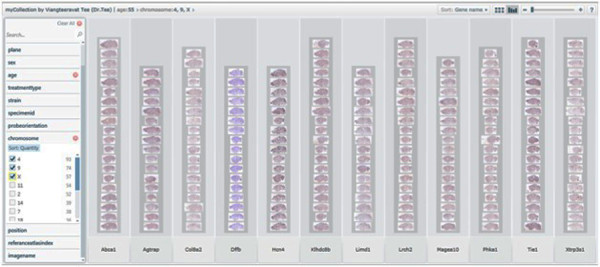
**Extracted genes based on categories**. Pivot collection showing Extracted genes based on categories.

We used a total of 1087 genes [8] that are co-expressed with Dab2 from the Allen Brain Atlas [8]. The different categories of genes used include gene name (see Table 1 below) and meta information for imageseriesid, plane, sex, age, treatmenttype, strain, specimenid, riboprobenam, probeorientation, position, imagedisplayname, referenceatlasindex to classify and cluster the genes to identify the hidden information relating to each of the meta information columns. Figure 9 shows a broad view of all the genes used. Figure 10 shows the classification of the gene based on the gene name. Figures 11, 12 and 13 show the images of expressions filtered with their associated data using Abca1, Klhdc8b and Pdlim4 genes respectively in the sagittal plane as a case study. Figure 14 shows the classification of the genes based on the chromosomes metadata, while Figure 15 shows that genes that are extracted when the sorting is based on gene name, chromosome (4, 9 and X) and at age 55. The same can be used to answer more complicated questions by applying more filters and conditions visually on the collection. And online access to the mouse brain pivot collection can be viewed using the link http://edtech-dev.uthsc.edu/CTSI/teeDev1/unittest/PaPa/collection.html (user name: tviangte and password: demome)

**Table 1:** Names and number of genes

Gene Name	Number
1810009M01Rik	148

2610528K11Rik	16

5430419D17Rik	18

A230063L24Rik	17

Abca1	20

Agtrap	17

BC054438	74

Calu	16

Cd63	119

Col8a2	19

Dffb	17

Dhx15	19

Efs	19

Enpp7	19

Gprin2	20

Hcn4	17

Id1	17

Kif13b	25

Klhdc8b	20

Limd1	17

Lrch2	20

Magea10	18

Mybl2	12

Notch2	19

Pak4	20

Pdlim4	18

Phka1	19

Rbm27	16

Sec24b	18

Slc22a8	80

Slc7a11	20

Smpdl3a	20

Sod3	76

Ss18	20

Tie1	20

Timd2	17

Xtrp3s1	20

**Total**	**1087**

Names of the 1087 genes used in the Pivot collection analysis.

## Discussion

This article has shown that with the necessary requirements like massive lab-imaging, an automated process for Pivot image collections can be generated. The Pivot collection is used in the clustering and classification of visual data into various deep zoom levels to detect the underlying hidden patterns within the data sets. This resource gives the user dynamic predictive ability with regard to the data items and also serves as a visualization tool. However, there are some limitations of the current Pivot implementation. The image analysis would typically be used by scientists and clinicians to examine research hypotheses that are defined with this current Pivot collection technology. There is also the expansion of the Analysis Tier to implement analytical and statistical functions which extracts meaningful information from Pivot collections such as an image marker to identify and share ROI, image feature comparison, and built-in basic statistical functions (e.g., t test, ANOVA, Correlation matrix) or an interface to statistical or imaging processing tool boxes (e.g., MATLAB), which will ultimately benefit the research community.

The middle layer service was written in PHP [30] to retrieve massive data sets along with metadata in XML format [31] from the Allen Brain Atlas using their proprietary API. The Pivot analysis provides users with ability to split, filter, and sort constraint variables and allows browsing at the different conceptual levels enabling mining processes, such as a decision tree mining. This enhances a key challenge to delivery of massive image collections of high-resolution images such as the Allen Brain Atlas projects and BrainMaps [16]. Pivot's user interface provides users with fast, interactive and intuitive online technologies to swiftly answer multi-dimensional, analytical queries from Pivot collections and support just-in-time resources and tools to be used to test the research hypotheses. For example, Figure 12 shows how genes are expressed in Chromosome 11 at any given age of the gene. Unlike other machine learning algorithms [32-34], which are restricted to a particular domain, our proposed algorithm/method can be applied to other biomedical domains like biomedical literature (text mining) and also within the geo-spatial domain [35] etc. In literature mining the text is converted into matrices to express words in sentence, while in geo-spatial mining, our algorithm can be used to identify the location (including the coordinates), age and sex of a given population; thus this is an algorithm with multiple functions and applications. With our ongoing development, we have no intention of reinventing advanced statistical software packages, such as SAS and image processing tools such as those in the MATLAB toolbox. On the contrary, we will develop a seamless interface to bridge Pivot collections with those software packages. We will provide users the ability to pre-process their biomedical images and neuro-imaging and then isolate extracted datasets for external examination (e.g., image-mining).

## Conclusions

Clinicians, laboratory researchers and other health-care providers generate a massive amount of neuro-imaging and clinical images in high resolution on daily basis; in fact, there is an "Information overload" [32] with the image collections being generated. Furthermore, the most common internet browsers now have improved browsing capabilities and network performance with respect to retrieving and downloading/uploading information from the internet/intranet or other online media. Thus it has become imperative for investigators of clinical projects to improve their method of image collection, to keep abreast and leverage the latest technology development in relation to image collection, collaboration, storing and transmission. Microsoft Live Labs Pivot technology empowers investigators of clinical research projects to interact with a massive amount of image collections seamlessly, thereby enabling them to analyze, filter, collaborate and share the image collections. The visual enhancement in the Microsoft Pivot technology makes data extraction and filtration very easy to use. We have proposed an automated process that will enable Microsoft Live Labs Pivot technology to create large sets of image collections.

Compared with any other data analysis technique like data mining, knowledge management and discovery, information and cognitive reasoning [34,36,37]; Azuaje [34] predicts cluster number from a given collection set without applying visual analytic component to the prediction making it difficult to identify the hidden information from the collection. Barbarane [36] evaluates cluster analysis solutions without identifying the hidden information in a data collection set. Badulescu [37] reviewed data mining algorithms used in clustering a data set collection but all the algorithms reviewed by Badulescu [37] does not visually analysed the data set to detect the hidden data/information. Pivot collection's visual analytics is used in identifying unexpected hidden information or data like strains of features that have been expressed in a gene database as shown in Figures 11, 12 and 13. Thus Pivot visual analytics is used in analyzing hard complex problems that other machine learning algorithms would otherwise find it difficult to analyze. It relieves the user from complex and sometimes complicated mathematical and statistical formulas associated with other machine learning methods and algorithms [33]. Our proposed algorithm has automated the creation of large image Pivot collections; this will enable investigators of clinical research projects to easily and quickly analyze the image collections with a view that is critical for making decisions about the image patterns discovered.

## Competing interests

The author declares that they have no competing interests.

## Authors' contributions

All the authors contributed equally to this work. All authors read and approved the final manuscript.
